# Quality of nursing work life and turnover intention among nurses of tertiary care hospitals in Riyadh: a cross-sectional survey

**DOI:** 10.1186/s12912-018-0312-0

**Published:** 2018-10-01

**Authors:** Bayan Kaddourah, Amani K. Abu-Shaheen, Mohamad Al-Tannir

**Affiliations:** 1 Ambulatory Care Centre, Executive Administration of Nursing Services, King Fahad Medical City, Riyadh, Saudi Arabia; 2 Research Center, King Fahad Medical City, Riyadh, Saudi Arabia

**Keywords:** QNWL, Nursing, Nurses’ turnover, Saudi Arabia

## Abstract

**Background:**

Nurse turnover has a negative impact on the ability to meet patient needs and provide a high quality of care, which may create more stress on other staff due to increased workloads. This can lead to critical changes in the behavior of nurses towards their jobs resulting in low work satisfaction, low productivity, and leaving the organization. Thus, this study aimed to assess the quality of nursing work life (QNWL), to explore the nurses’ turnover intention and to examine the correlation between QNWL and nurses’ turnover intention.

**Methods:**

A cross-sectional survey was conducted on nurses with at least 1 year of nursing experience at two hospitals selected randomly from Riyadh, Saudi Arabia: King Fahad Medical City and King Faisal Specialized Hospitals. Data were collected using a self-administered questionnaire comprising four sections (Brooks’ survey of QNWL, Anticipated Turnover Scale (ATS), open-ended questions and demographic characteristics).

**Results:**

A sample of 364 nurses was recruited. Results proposed that the participants were dissatisfied with their work life (54.7%), with almost 94% indicating a turnover intention from their current hospital. Moreover, 154 (93.3%) out of 165 nurses who reported satisfaction with QNWL indicated the intention to turnover. The correlation between QNWL and ATS for binary variables was too week (*r* = − 0.024) and statistically not significant (*p* = 0.206).

**Conclusion:**

The QNWL and nurse turnover are challenging issues for healthcare organizations because of its consequences and impact on patient care. Our study provided critical findings low indication satisfaction of nurses with their QNWL and a high turnover intention. The results of this study could be used as a nexus for the development of regulations and practical strategies to enhance QNWL and to decrease the turnover.

## Background

Health organizations in many countries have faced some difficulties like shortage of health experts, and increase the turnover rate, especially amongst nurses. Nurse turnover has a negative impact on the ability to encounter the patient needs and deliver high standards of care [1]. Additionally, the turnover of nurses leads to insufficient staffing, which increases the workloads and stress on other staff [2–4]. Consequently, this may lead to serious variations in nurse’s behavior towards their jobs causing low work satisfaction and productivity, and then shifting to another organization. As well, insufficient nurse staffing leads to poor patient outcomes, like increased patient mortality and infection rates error rates might be increased [5, 6]. The previous research in the health field has concentrated on answering these issues and their related causes [4, 5]. Some studies have shown the impact of quality of work life on the commitment of nurses and other health professionals [4, 7, 8]. The quality of nursing work life (QNWL) defined as the degree to which registered nurses can satisfy important personal needs through their experiences in their work organization while achieving the organization’s goals [9]. Evaluating QNWL permits organizations to realize how challenges in work environments affect nurse’s job satisfaction and commitments [10]. The high quality of work life is crucial for organizations to appeal to new employees and maintain their workforces. However, reliable information on the quality of work life and turnover intention of nurses is limited in Saudi Arabia. In an in-depth review of literature proposes that there is a remarkable increase in the rate of nurse organizational turnover supplemented via an insignificant increase in nursing resource [7, 9]. In Saudi Arabia, nurses come from different countries, some of which may be unharmonious with the culture of Saudi Arabia. The previous study reported that non-Arabic speaking nurses are at a shortcoming as care providers for people in public because of the language barrier and cultural variances [11]. Such challenges for nurses alongside other causes linked to the organization generate a high level of stress which adversely affects the quality of their working life. Another study conducted by Alhusaini and Alamri et al. indicated that the majority of foreign nurses move to industrialized countries after attaining enough experience [12, 13]. In consequent of, competition to attract competent nurses, Saudi Arabia might miss experienced nurses who may choose to work with other establishments that deliver right working environments. It is integral for a policymaker to assess their quality of work life and to understand their organizational and career intentions, which may improve the nurses’ work satisfaction and productivity, which subsequently improve the health services being delivered to their patients. Thus, the objectives of this study were to assess the QNWL, to explore the nurses’ turnover intention and to examine the correlation between QNWL and nurses’ turnover intention.

## Methods

### Study design and settings

A cross-sectional survey was conducted at two out of four tertiary hospitals in Riyadh city, Saudi Arabia. The hospitals were selected randomly: King Fahad Medical City (KFMC) and King Faisal Specialized Hospitals (KFSH) from March 2015 to March 2016.

### Study participants and sampling

Nurses with at least 1 year of nursing experience in various clinical settings at KFMC and KFSH were eligible to participate in this study. Nurses working at different shifts were selected randomly from the two hospitals by a quota-based sample, after obtaining written informed consent form from each participant. Data were collected by administering self-reported questionnaires in the English language as it is the main using language for the staff in the hospital and they studied nursing in the English language.

Participants were provided with the study package which included the cover letter, questionnaire, and an envelope. Moreover, participants were received a reminder by the study coordinator to fill and put the completed survey in a large labeled envelope and then return them to the hospital mail service ensuring anonymity and confidentiality.

### Data measurement

Two instruments were used to answer the objectives of this study: Brooks’ survey of QNWL and anticipated turnover scale (ATS). The developers of the research instruments granted permission to use their questionnaires.

The QNWL is a self-administered questionnaire which encompasses 42 items in four subscales which are work life/home life, work design, work context and work the world. The work life/home life dimension is the interface between the nurses’ work and home life. The work design dimension is the composition of nursing work and describes the actual work that nurses perform. The work context dimension includes the practice settings in which nurses’ work. Finally, the work world dimension is defined as the effects of broad societal influences and changes in the practice of nursing [14]. The instrument asks respondent nurses how much they agree or disagree with each item on a 6-point scale, ‘1′ indicating ‘strongly disagree’ and ‘6′ indicating ‘strongly agree’. Responses with means ≤4.2 were considered as an indication of dissatisfaction of respondents towards the quality of work life. The reliability of the scale is 0.90 and construct validity is 0.89 [9].

The ATS is a 12-item self-administered questionnaire. It was created by Hinshaw and Atwood to investigate the turnover intention amongst nurses. It measures the employees’ attitudes and perceptions of the probability of terminating their current job [15]. The ATS instrument is a 7-point Likert scale ranging from ‘agree strongly’ to ‘disagree strongly’ [15]. The instrument’s items were related to an employee’s anticipated length of time to leave and certainty of leaving the job. The total score was obtained by calculating the sum of all items in the scale divided by the number of items in the scale. Greater scores reveal a more intent to leave the current job. Responses with means > 3.5 were reflected as a sign of turnover intention [16]. The internal consistency reliability estimated with Cronbach’s α was 0.84 [17].

As well as, the QNWL and ATS scales; demographic data (age, gender, marital status, family members living in KSA, having children, primary family caregiver, total years of nursing experience, total years of nursing experience in Saudi Arabia, current nursing position, total years working in the current position, monthly income, having additional financial benefits) which may affect the findings were also collected from the study participants. Demographic characteristics exhibit independent effects: therefore, they must be considered as contributors to turnover [18].

The study assumes a correlation between work-life related factors, nurses’ demographic characteristics, quality of work life (QWL) level and turnover intention. QWL is influenced by work-life factors as reported by Brooks and Anderson [9] as well as demographic factors, which might lead either too high or low level of QWL, which can seriously change the behavioral intention of the nurses.

### Pilot study

The pilot study was conducted to test the clarity and time taken to fill out the questionnaire. Seventeen (*N* = 17) nurses from one hospital participated. According to the participants’ recommendations some modifications were made to the questionnaires. Based on the pilot study, the results showed that for QNWL and its dimensions, Cronbach’s’ α were 0.89 for QNWL and 0.90 for the ATS.

### Sample size estimation

The sample size was calculated using the Raosoft online sample size calculator with 5% margin of error and a 95% confidence interval. Based on results from the pilot study, the prevalence of turnover intention was estimated at 32%, which allowed us to calculate the needed sample size of 365 subjects.

### Statistical analysis

All data was entered and analyzed through statistical package SPSS version 22. Categorical variables were presented in frequencies and percentages. Whereas, continuous variables like all test scores ATS and QNWL were expressed as mean (S.D). Cronbach α test was applied to assess the internal consistency of QNWL and ATS. Independent sample t-test/ANOVA was used to determine the mean score of ATS and QNWL with demographic characteristics of the survey’s participants. A bivariate stepwise logistic regression analysis was performed to identify the factors that might be associated with ATS score. *P*-value < 0.05 two-tailed was considered as statistically significant.

## Results

A representative sample of 400 nurses was invited to participants in this study, out of whom 364 nurses were recruited (response rate is 91%). Respondents’ demographic characteristics were presented in Table 1. A total of 329 (90.4%) of respondents were females. Two hundred thirty 236 (64.8%) nurses are married, and 149 (40.9%) respondents had family members living in KSA. The majority of participants 198 (54.5%) had between 1 to 5 years of clinical experience in Saudi Arabia and were Filipino 206 (56.6%).

**Table 1:** Demographic characteristics of respondents

Characteristics	Categories	*n* (%)
Gender	Female	329 (90.4)
	Male	35 (9.6)
Age Group	22–30	179 (49.2)
	31–40	132 (36.3)
	41–50	45 (12.4)
	> 51	8 (2.2)
Marital Status	Married	236 (64.8)
	Single	122 (33.5)
	Widowed	3 (0.8)
	Divorced	3 (0.8)
Nationality	Filipino	206 (56.6)
	Indian	106 (29.1)
	Saudi	9 (2.5)
	Jordanian	7 (1.9)
	others	36 (9.9)
Family members living in KSA	Yes	149 (40.9)
	No	215 (59.1)
Having children	Yes	162 (44.5)
	No	202 (55.5)
Primary family caregiver	Yes	103 (28.3)
	No	261 (71.7)
Education level	Associate Degree	5 (1.4)
	Diploma	81 (22.3)
	Bachelor	275 (75.5)
	Master’s Degree	3 (0.8)
Total years of nursing experience	1–5	88 (24.2)
	6–10	58 (15.9)
	11–15	149 (40.9)
	> 15	69 (19)
Total years of nursing experience in Saudi Arabia	1–5	198 (54.5)
	6–10	42 (11.5)
	11–15	100 (27.5)
	> 15	24 (6.6)
Current nursing position	Staff Nurse	334 (91.8)
	Charge Nurse	25 (6.9)
	Others	5 (1.4)
Total years working in the current position	≤5	201 (58.5)
	> 5	151 (41.5)
Monthly income (SR)	< 5000	200 (54.9)
	5000–10,000	153 (42)
	11,000 – 15,000	8 (2.2)
	> 15,000	3 (0.8)
Having additional financial benefits (e.g. housing allowance)	Yes	151 (41.5)
	No	213 (58.5)
ATS cutoff	< 3.5	22 (6.0)
	> 3.5	342 (94)
QWL Cutoff	< 4.2	199 (54.7%)
	> 4.2	165 (45.3%)

*SR* Saudi Riyal

According to ATS criteria of cutoff (> 3.5) intent to leave the current position, our data shows that 94% of participants were willing to leave the current job. Moreover, QNWL criteria of cutoff (≤ 4.2) satisfaction towards the QWL revealed that 54.7% of participated nurses were dissatisfied with the quality of work life. There is no significant difference between the answers given by the respondent’s nurses were working in different departments in the two different hospitals.

Table 2 shows the association of demographic characteristics with the cut off levels of the QNWL and ATS scales. The majority of male nurses 21 (60%) were satisfied with the QNWL than females 144 (43.8%). Among the age group, the majority of nurses were dissatisfied with the QNWL except in the age group 31–40 years. Moreover, about 75 (61.5%) of the never married nurses were dissatisfied with the QNWL compared to 120 (50.8) who were married. The majority of nurses with associate degree 4 (80) and those with less than 5 years of experience were reported dissatisfaction with QNWL. Nurses with a monthly income of more than 15,000 SAR 3 (100%) were all satisfied with QNWL. Overall, a significant difference was found between satisfactory nurse status and total years of nursing experience (*p* = 0.025), total years of nursing experience in Saudi Arabia (*p* = < 0.001), and monthly income (*p* = 0.005). However, further logistic regression analysis showed that nurses who have been working in Saudi Arabia between 11 and 15 years were the only satisfied (*p* = 0.006) with QNWL among other nurses. Also, nurses with monthly income less than 5000 and 5000–10,000 Saudi Riyal were found to exhibit an effect on nurses’ satisfaction (*p* = < 0.001; for both) about QNWL.

**Table 2:** Association of demographic characteristics with QNWL and ATS

		QNWL			ATS		
		< 4.2 *n*(*n*)	≥ 4.2 *n*(*n*)	*p* value	≤ 3.5 *n*(*n*)	> 3.5 *n*(*n*)	*p* value
Gender	Male	14 (40)	21 (60)	0.067	5 (14.3)	30 (85.7)	0.031*
	Female	185 (56.2)	144 (43.8)		17 (5.2)	312 (94.8)	
Age Group	22–30	107 (59.8)	72 (40.2)	0.071	8 (4.5)	171 (95.5)	0.196
	31–40	61 (46.2)	71 (53.8)		12 (9.1)	120 (90.9)	
	41–50	25 (55.6)	20 (44.4)		1 (2.2)	44 (97.8)	
	> 51	6 (75)	2 (25)		1 (12.5)	7 (87.5)	
Marital Status	Divorced	2 (66.7)	1 (33.3)	0.259	0 (.0)	3 (100.0)	0.218
	Married	120 (50.8)	116 (49.2)		15 (6.4)	221 (93.6)	
	Single	75 (61.5)	47 (38.5)		6 (4.9)	116 (95.1)	
	Widowed	2 (66.7)	1 (33.3)		1 (33.3)	2 (66.7)	
Dependent Children	Yes	91 (56.2)	71 (43.8)	0.606	8 (4.9)	154 (95.1)	0.428
	No	108 (53.5)	94 (46.5)		14 (6.9)	188 (93.1)	
Education Level	Associate Degree	4 (80)	1 (20)	0.682	1 (20.0)	4 (80.0)	0.252
	Bachelor Degree	149 (54.2)	126 (45.8)		19 (6.9)	256 (93.1)	
	Diploma	44 (54.3)	37 (45.7)		2 (2.5)	79 (97.5)	
	Master’s Degree	2 (66.7)	1 (33.3)		0 (.0)	3 (100.0)	
Total years of nursing experience	1–5 years	60 (68.2)	28 (31.8)	0.025*	4 (4.5)	84 (95.5)	0.706
	11–15 years	28 (48.3)	30 (51.7)		4 (6.9)	54 (93.1)	
	6–10 years	73 (49)	76 (51)		8 (5.4)	141 (94.6)	
	> 15	38 (55.1)	31 (44.9)		6 (8.7)	63 (91.3)	
Total years of nursing experience in Saudi Arabia	0–5 years	122 (61.6)	76 (38.4)	< 0.001*	8 (4.0)	190 (96.0)	0.112
	6–10 years	43 (43)	57 (57)		11 (11.0)	89 (89.0)	
	11–15 years	15 (35.7)	27 (64.3)		2 (4.8)	40 (95.2)	
	> 15	19 (79.2)	5 (20.8)		1 (4.2)	23 (95.8)	
Current nursing position	Charge Nurse	13 (52)	12 (48)	0.504	2 (8.0)	23 (92.0)	0.782
	Staff Nurse	182 (54.5)	152 (45.5)		20 (6.0)	314 (94.0)	
	Other	4 (80)	1 (20)		0 (.0)	5 (100.0)	
Total years working in the current position	0–5 years	123 (57.7)	90 (42.3)	0.161	6 (2.8)	207 (97.2)	0.002*
	> 5 years	76 (50.3)	75 (49.7)		16 (10.6)	135 (89.4)	
Monthly income (SR)	< 5000	124 (62)	76 (38)	0.005*	7 (3.5)	193 (96.5)	0.079
	5000–10,000	70 (45.8)	83 (54.2)		15 (9.8)	138 (90.2)	
	11,000–15,000	5 (62.5)	3 (37.5)		0 (.0)	8 (100.0)	
	> 15,000	0	3 (100)		0 (.0)	3 (100.0)	

(*) Shows that *p*-value is significant

Regarding the anticipated turnover intention, the majority of males 30 (85.7%) and females 312 (94.8%) have the intention to turnover. Moreover, the vast majority of nurses at their different demographic characteristics have reported the intention to turnover. Also, the statistically significant difference was only found between nurse intention to turnover with gender (*p* = 0.031) and total years working in the current position (*p* = 0.002). Nevertheless, logistic regression analysis showed that charge nurse position (*p* = < 0.001) and total years working in the current position (*p* = 0.017) were found to exhibit an effect on nurses’ intention to turnover (Table 2).

Table 3 shows that among the 199 nurses who were dissatisfied with the QNWL, about 188 (94.5) of them have the intention to turnover. Moreover, 154 (93.3) out of 165 nurses who reported satisfaction with QNWL indicated the intention to turnover. The correlation between QNWL and ATS for binary variables was a week (*r* = − 0.024) and statistically not significant (*p* = 0.206).

**Table 3:** Correlation between QNWL scale and ATS scale

		Cut off Value of QNWL			*p*-value
		< 4.2	≥ 4.2	Total	
Cut off Value of ATS	≤ 3.5	11(5.5)	11(6.7)	22(6.0)	0.206
	> 3.5	188(94.5)	154(93.3)	342(94.0)	
	Total	199(54.7)	165(45.3)	364(100.0)	

## Discussion

This study provides an initial step in understanding the work life of nurses in tertiary care sitting and shed light on an important topic which if not assessed and well managed by an organization might have a negative impact on the ability to meet the patient needs and deliver high standards of care. Our study revealed that the majority (54.7%) of respondents was dissatisfied with their work life and 94% have turnover intention. Our result is higher than reported in previous studies on nurses’ turnover intention. A study carried out by Brooks et al., to assess the QNWL of staff nurses using the same scale reported nursing dissatisfaction with their work life [17]. In Saudi Arabia, a study conducted by Almalki et al., on 508 primary health care nurses, reported that the participants were dissatisfied with their work life, with nearly 40% showing a turnover intention [19]. In Sweden, a prospective study on 1417 nurses showed that every fifth nurse intended to leave their profession after 5 years [20]. In a study based on 6469 hospital nurses in seven European countries, reported that nurses have reward frustration with higher turnover intentions [21]. Our results were critical and alarming, however, these findings could be explained by the fact that the majority of the nurse in the study sites were expatriate nurses from different countries.

Previous studies reported monthly income, length of work experience, and organization tenure as important predictors of employees’ satisfaction with QWL [22, 23]. Our results showed that total years of nursing experience, total years of nursing experience in Saudi Arabia, and monthly income significantly affect nurses’ satisfaction about QNWL. This could be explained by that nurses with more work experience have more stability in their job and thus experience a better QNWL. However, previous studies conducted by Nayeri et al. and Boonrod revealed that they could not found a significant association between QNWL and the length of work experience [24, 25].

Consistent with previous studies our results showed that gender, total years working in the current position were also affecting nurses’ intention to turnover [17, 24]. While, other studies reported no relationship between gender and employees’ intention to leave their work [25, 26].

Although previous studies confirmed the significance of family to the view of the nurses to their QNWL [1, 17], the results of the present study showed no significant association between QNWL and neither family member living in KSA nor marital status. Similarly, two studies have also found that QWL has no significant relationship with marital status [23, 24].

In the present study we examined the correlation between QNWL and ATS. Although the work context dimension makes the strongest contribution to explaining turnover intention, our results showed no correlation between QNWL and ATS. On the contrary, prior studies reported that the intention to turnover could be explained by the nurse’s satisfaction with QNWL [27, 28]. In previous studies based on 1283 hospital nurses in Taiwan and 740 nurses in Iran reported that nurses’ QWLs were significantly negatively associated with nurses’ turnover intentions [29, 30].

Efficacious QNWL strategies in healthcare settings can enhance employees’ self-esteem and organizational efficiency. Furthermore, QNWL can advance the quality of care provided in addition to staffing and preservation of the nursing workforce. This high rate of turnover intention ought to motivate the nursing leaders to develop appropriate and efficient strategies to combat this serious issue and improve the nurses work conditions and their QNWL, which consequently, will enable the nurses to perform better care for their patients.

The strengths of the study that it can be generalizable to the nursing staff working at tertiary hospitals in Riyadh city as the study was conducted at two out of four reputable tertiary hospitals in Riyadh city, Saudi Arabia. Furthermore, the possibility of non-response bias is very little as the response rate is high (91%). While, the limitations that the study is a cross-sectional and data collected using self- administer scales. Therefore, further studies should be carried out with more objective instruments.

Further studies are needed to understand and improve nurses’ QNWL and retention. It would be valuable to conduct longitudinal and interventional studies to assess the definite turnover amongst nurses paralleled with described turnover intention in the present study. Additional an in-depth study is required to explore the effect of social and cultural norms on the perception of expatriate nurses towards QNWL and turnover intention.

## Conclusions

The QNWL and nurse turnover are challenging issues for healthcare organizations because of the consequences and impact on patient care. Our study provides critical findings low indication satisfaction of nurses with their QNWL and a high turnover intention. The results of this study could be used as a guide for the development of regulations and practical strategies to enhance QNWL and to decrease the turnover.

## Abbreviations

ATS: Anticipated Turnover Scale
KFMC: King Fahad Medical City
KFSH: King Faisal Specialized Hospitals
QNWL: Quality of Nursing Work Life
QWL: Quality of Work Life
